# Regarding “A Cluster-Randomized Trial to Test Sharing Histories as a Training Method for Community Health Workers in Peru”

**DOI:** 10.9745/GHSP-D-21-00178

**Published:** 2021-06-30

**Authors:** Daniel C. Taylor

**Affiliations:** a Future Generations University, Franklin, WV, USA.

## Abstract

Improving communication between mothers and health systems will grow cost-effective, potentially scalable health impact. By developing an approach of how health systems and mothers can communicate to increase mutual understanding, a "health language" that is grounded in mothers' reproductive life narratives can be developed to help bridge the long-standing gap in how health systems and mothers engage.

See related article by Altobelli et al.

## THE NEED FOR UNDERSTANDABLE LANGUAGE IN HEALTH CARE DELIVERY

The article by Altobelli et al.^1^ has implications beyond the important study they describe. The authors have done a superb job of explaining their research and providing robust statistical evidence to support it. There is no need to review here their well-written description, given the breakthrough import of their research. Instead, I want to point out the implications of this article in terms of *how the language of mothers offers now-not-utilized potential in health systems delivery.* Language is not normally considered fundamental to health delivery, but I suggest here that this cluster-randomized trial shows that language is foundational.

The need for effective language is starkly shown in how health workers often speak of mothers as being “misinformed” or even “illiterate.” In contrast, mothers say, “my doctor doesn't get it.” This bidirectional misunderstanding doesn't answer either's needs. If clients and their health providers cannot understand each other, how can they work together? But Altobelli et al.^1^ show that if health care workers use understandable language, they can more effectively partner with (and not just “target”) humanity's number one health care provider: *mothers*. Health systems can be rooted in the health's place of grounding: *homes*.

This implies that health providers and health facilities are everywhere. Health delivery's long-pursued quest for the “last mile-to-the-home” would be finally attained. Informed health delivery can potentially start everywhere and be informed by dialogue with the health system.

## BRIDGING THE LANGUAGE GAP BETWEEN MOTHERS AND HEALTH SYSTEMS

Altobelli et al.^1^ suggest that a language exists in mothers' narratives of their reproductive life experiences. Since the dawn of speech, mothers have talked about health. As mothers' language continually validates itself as their children survive, humanity added a parallel language of health systems delivery—framed by science that seeks to be universal and often holds a male bias. Because of its scientific and masculine nature, this language created a gap with the feminine language that has been used in successfully raising our species. Let us close this gap.

In all candor, both languages are incomplete. Why not have health systems learn the language that already exists? Health providers long ago learned that they should drop Latin medical vocabulary. Their next task is to expand health science syntax so it affirms the experiences of mothers. To achieve this, mothers do not need to learn health science, nor do health professionals need to morph into anthropologists. But they must both translate their languages, and thereby open them to each other. This is what Sharing Histories has the potential to grow into: a “Rosetta Stone” that can help in the understanding of family health.

## SHARING HISTORIES ENABLES BIDIRECTIONAL LEARNING AND UNDERSTANDING

Altobelli et al.^1^ show that more effective training of community health workers (CHWs) supplies the needed link between these systems of communication. Sharing Histories allows mothers to better understand health, as CHWs explain science using the names of children they know. On the other side, health systems get local hard evidence specific to the distinct features of each community.

How is this bidirectional understanding discovered? The process is based on the now-established universality by which women remember their reproductive histories with acute accuracy and self-understanding that is foundational to their identities. Mothers share such life experiences with each other as their children grow, forming a narrative with their children's names. The opportunity is to use this narrative to structure a better understanding of each community. After women present their lived experiences, their experiences that each mother has already memorized through knowing the child are informed by health science using the bidirectional process.

A group of approximately 20 mature mothers, who each represent a community, are led by a CHW from the health system in a confidential discussion. Each mother narrates her reproductive history in a “distilled Demographic and Health Survey.”

At regular intervals, a common theme in the narratives is explained that is specific to a child. Understand this process in this way: the life narratives are like photographs in a science book where the photo captions have the children's names and also now an understandable scientific explanation of their health.

The CHW explains actions that mothers can take to treat these conditions. That is, this group of mothers learns how to care for the sick and support each other. By learning, these mothers become better care providers for their families; *more importantly, they become accepted basic care providers in their communities.*

Creative planning can grow for how community systems can be engaged to address needs, such as sanitation or violence toward women. Women are supported to be proactive in addressing the determinants of health in other sectors.

A continuing framework is launched to support these groups. Their structure must fit national and local policies, and this occurs by their engagement through the CHW structure.

Baseline information is collected during the first gathering. During subsequent meetings, community change is tracked, and from continuing meetings, ongoing evidence is accrued (with the exciting consequence of gaining community-specific health data).

This system allows mothers (through basic care and prevention) to engage with the health system for the majority of their health events. Health knowledge has become localized, which helps launch continuing action by the whole community.

Several studies have shown a woman's narrative of her reproductive history from her first menstrual period forward is memorized evidence. Our study^2^ from our university's work in Afghanistan, which preceded Altobelli et al., supports this. Aggregated across women of a community, a “medical textbook” can be created that is specific to that community. This is a data set of what collectively happened to that group—evidence known by all women. If this is then understood by health systems, localized health delivery can be more effective. Mothers talking about their children, especially their experiences of having children, functions as a language of shared understanding contextualized to them.

Usually, such information is dismissed by those in the health system as simply “women's stories.” But, linguistically, through Sharing Histories, this can become a dialect where the women in every group know the names of important health conditions—that is, words describing whole health experiences. So, simply mentioning those names speaks of that community's health evidence. Typically, these are discussions known only to that group of mothers. The Altobelli et al.^1^ article highlights how this communication that is usually only within a closed group can inform formal health systems. These communications-based maternal experiences allow mothers to grasp health practices and allow health systems to understand local realities. The result is that health systems can function more effectively.

## EMPOWERING MOTHERS TO PARTNER WITH HEALTH SYSTEMS

This article shows us the way to advance beyond where health systems view mothers as “misinformed,” or “illiterate.” Health providers can learn from a data set of “stories.” On the other hand, mothers need no longer say, “my doctor doesn't get it.” Health science can be explained using names they have memorized with the corresponding conditions. By closing that gap of misunderstanding, an empowered partnership is formed.

The empowerment of mothers is widely held as the ultimate answer to health action—delivering reach to the unreached. Achieving mothers' empowerment has been, until now, mostly a “black box.” The Sharing Histories method opens this box by coupling women's experiential knowledge with scientific understanding.

Once women can communicate more effectively with health service providers, other applications of this method, such as in India and Afghanistan, have shown that engagement can broaden to other development services, such as livelihoods, education, governance, and food security.^2^ What could result is confidence in the formal health system and could allow engagement beyond with society enabling systems that are the determinants of health. Health could grow from inside communities, and this growth could be informed from outside by knowledge and supervision that mothers understand. As this article^1^ shows, this language can be taught to mothers by CHWs and can become a modality of communication with health and social systems.
